# Mass treatment of tapeworms for *Taenia solium* eradication

**DOI:** 10.1371/journal.pntd.0013762

**Published:** 2025-12-03

**Authors:** A. Clinton White

**Affiliations:** Infectious Disease Division, Department of Internal Medicine, University of Texas Medical Branch, Galveston, Texas, United States of America; University of Washington, UNITED STATES OF AMERICA

In 2021, the PanAmerican Health Organization reviewed evidence and made recommendations in favour of preventive treatment for *Taenia solium* tapeworm carriers as part of efforts to eradicate neurocysticercosis [1]. The recommendations suggested that either praziquantel or niclosamide should be the main agent used for human tapeworm carriers. Albendazole was recommended only as an alternative if the main treatments were not available.

At that time, there were many uncertainties regarding the recommendations. Niclosamide is approved for treatment of tapeworm infections including *T. solium*. However, in 2021, there were limited data on its safety and efficacy in mass chemotherapy. Wardel and colleagues have now reported on the safety and efficacy of niclosamide in mass chemotherapy of 68,751 adults and children >2 years old in northern Peru [2]. Overall, there were no serious adverse events. Only 1.5% reported any adverse events at a 72 h follow-up visit. None of the adverse events were severe and 99.2% were reported as mild. In terms of efficacy, 211 were diagnosed with taeniasis (all due to *T. solium*). At 30-day follow-up, 75% were cured. The cure rate was assessed by both microscopy and a sensitive antigen-detection test. Thus, this study may have underestimated efficacy compared to other studies.

There were reports that praziquantel could induce symptoms of neurocysticercosis. However, the data were limited on how frequently this occurs and how to safely manage this. Nelly and colleagues now report on the results of 2 mass treatment campaigns conducted in Madagascar [3]. Prior to these campaigns, there was a report of a death during mass treatment that was attributed to neurocysticercosis. Thus, prior to the campaigns, the authors designed a method of screening all participants for symptoms suggestive of neurocysticercosis. Those screening positive were treated with niclosamide instead of praziquantel. The study also provided active screening for symptoms at 3 days of follow-up and passively for 7 days. They provided a referral mechanism for those with symptoms at either time point.

During the first round of treatment, 116,601 individuals were treated with praziquantel and 615 were treated with niclosamide [3]. In the second round, 162,106 were treated with praziquantel and 983 were treated with niclosamide. In the first round, there was 1 serious adverse event. In the second round, there were 9 serious adverse events, mainly seizures. With symptom screening and treatment with niclosamide for those with symptoms, 10/278,707 developed serious adverse events; seizures occurred in <0.004% of those treated. Among those with seizures who underwent CT scans, 2 of 5 showed lesions consistent with neurocysticercosis, as did 1/3 who developed severe headaches. Similar results were noted in a trial in Zambia in which patients were screened for serum antigen, using niclosamide in those who were antigen positive [4]. Thus, with careful screening and available neurologic treatment, praziquantel can be safely used in mass chemotherapy to eradicate *T. solium* taeniasis. Both studies stated that praziquantel was thought to be more effective than niclosamide. Whether this is true is not known. The methods used in studies of praziquantel efficacy were not as rigorous as those used with niclosamide and likely significantly overstated efficacy. For example, Bustos and colleagues demonstrated that patients treated for tapeworms with negative stool studies can be demonstrated to have treatment failure by antigen-detection and retreatment [5]. Lower efficacy of praziquantel has been noted in other field studies [6].

The third drug, only recommended as an alternative, was albendazole. The doses of the drug used for tapeworms are similar to doses used for treatment of neurocysticercosis. Also, the duration of treatment was 3 days (as opposed to single doses for praziquantel and niclosamide). There are anecdotes of albendazole treatment causing symptoms in latent neurocysticercosis. More recently, Modingam and colleagues reviewed the WHO pharmacovigilance database for serious adverse events from benzimidazoles compared to other antiparasitics [7]. They confirmed the known association of benzimidazoles with hepatitis and blood dyscrasias. However, they also noted that benzimidazoles are more frequently associated with seizures and headaches. Thus, this larger database confirmed concerns about the safety of albendazole in areas endemic for *T. solium*.

Overall, these studies have confirmed the wisdom of the PAHO/WHO experts. Both praziquantel and niclosamide can be used safely in mass treatment for *T. solium* eradication. In the case of praziquantel, however, this requires screening for either symptoms or antigen suggestive of cysticercosis. Niclosamide has a better safety profile and can be safely used in endemic areas without screening. There are no good data on relative efficacy of the 2 drugs. By contrast, albendazole appears to be much less advantageous. It requires a multiday dosing schedule and there is increasing concerns about serious neurologic adverse events.
